# An Open Retrospective Study of a Standardized Cannabidiol Based-Oil in Treatment-Resistant Epilepsy

**DOI:** 10.1089/can.2019.0082

**Published:** 2022-04-19

**Authors:** Francesca Marchese, Maria Stella Vari, Ganna Balagura, Antonella Riva, Vincenzo Salpietro, Alberto Verrotti, Rita Citraro, Simona Lattanzi, Carlo Minetti, Emilio Russo, Pasquale Striano

**Affiliations:** ^1^ Department of Neurosciences, Rehabilitation, Ophthalmology, Genetics, Maternal and Child Health, University of Genoa, Genoa, Italy.; ^2^ Paediatric Neurology and Muscular Disease Unit, IRCCS “Giannina Gaslini” Institute, Genoa, Italy.; ^3^ Department of Pediatrics, University of L'Aquila, L'Aquila, Italy.; ^4^ Department of Science of Health, School of Medicine and Surgery, University “Magna Graecia” of Catanzaro, Catanzaro, Italy.; ^5^ Neurological Clinic, Department of Experimental and Clinical Medicine, Marche Polytechnic University, Ancona, Italy.

**Keywords:** cannabidiol, drug resistance, epilepsy, treatment

## Abstract

**Introduction:**

Cannabidiol (CBD) has antiseizure properties but no psychoactive effects. Randomized controlled trials of an oral, pharmaceutical formulation of highly purified CBD are promising; however, data regarding other formulations are sparse and anecdotal. We evaluated the effectiveness of add-on therapy with a standardized CBD-based oil in treatment-resistant epilepsy (TRE) patients.

**Materials and Methods:**

An open retrospective study was carried out on patients with refractory epilepsy of different etiology. We reviewed clinical data from medical charts and caregiver's information. Participants received add-on with 24% CBD-based oil, sublingually administered, at the starting dose of 5–10 mg/[kg·day] up to the maximum dose of 50 mg/[kg·day], based on clinical efficacy. Efficacy was evaluated based on patients being seizure free or experiencing at ≥50% improvement on seizure frequency. Tolerability and suspected adverse drug reaction data were also analyzed.

**Results:**

We included 37 patients (46% female) with a median age of 16.1 (range: 2–54) years. Twenty-two (60%) patients suffered from epileptic encephalopathy, 9 (24%) from focal epilepsy, and 6 (16%) from generalized epilepsy. Mean follow-up duration was 68 (range: 24–72) weeks. The average age at seizure onset was 3.8±2.1 years (range: 7 days–21 years). The median achieved CBD-based oil dose was 4.2±11.4 (range: 0.6–50) mg/[kg·day]. At 40-month follow-up, 7 (19%) patients were seizure free, 27 (73%) reported >50% improvement, 2 (5%) patients reported <50% improvement, and 1 patient discontinued therapy due to lack of efficacy. Weaning from concomitant antiepileptic drugs was obtained after 24 weeks from CBD introduction in 10 subjects. Mild and transitory adverse events, including somnolence or loss of appetite, occurred in nine (25%) patients.

**Discussion and Conclusion:**

We showed the efficacy of a CBD-based oil formulation with few significant side effects in patients with TRE of various etiologies.

## Introduction

Epilepsy is one of the most common chronic disorders of the brain affecting around 70 million people worldwide. Treatment is mainly symptomatic, and most individuals have a favorable prognosis and achieve long-term seizure control.^1^ However, almost one-third of the patients' results show resistance to anticonvulsant therapy. Pharmacoresistance is defined by the failure to reach a complete or acceptable control in response to antiepileptic drugs.^2,3^ Despite the increasing number of available treatments, including drugs, neuromodulation, and surgical and dietary interventions, the burden of treatment-resistant epilepsy (TRE) has remained stable over the years, and the search for alternative and adjunctive therapies is likely to be of great interest.^4,5^

The effectiveness of cannabis-derived products in the treatment of epilepsy has been reported since antiquity,^6,7^ and they have been part of pharmacopoeia in the United States until the 1930s.^8^ The cannabis plant contains more than 100 phytocannabinoids that can influence the human body through several mechanisms.^9,10^ Among these, the main phytocannabinoids are delta-9 tetrahydrocannabinol (THC) and cannabidiol (CBD), which are present in the plant in high concentration. In contrast to THC, CBD is a nonpsychoactive/psychotropic substance according to its lack of effects on cannabinoid receptors type 1 and 2.^11^

Recent data reported in the literature^12–14^ showed that CBD has good antiseizure activity in a broad range of epilepsy syndromes and etiologies although its mechanism of action has not yet been completely clarified.^15,16^ However, the efficacy of CBD may vary by epilepsy syndrome, seizure type, age, or route of administration. Bioavailability of oral CBD is around 10%, whereas the sublingual bioavailability is at least ≥80%.^17–19^ Moreover, CBD is thought to have other properties such as anti-inflammatory, neuroprotective, and antioxidant.^7^

Randomized placebo-controlled trials have demonstrated that an oral, standardized pharmaceutical formulation of highly purified CBD (Epidyolex^®^) is a tolerable and effective treatment for two severe childhood-onset epilepsies, Dravet Syndrome (DS) and Lennox–Gastaut syndrome (LGS).^11,19,20^ Several open-label expanded access programs have been initiated to study this pharmaceutical formulation of CBD for the treatment of TRE in patients with other seizure etiologies, but to date, there are limited data on other epilepsy syndromes, including many of the severe genetic epilepsies.^9,21–26^ We evaluated the effectiveness of add-on treatment with a 24% CBD-based oil in patients with TRE.

## Methods

### Patients

This was an open-label retrospective study on patients with refractory epilepsy of different etiology who received add-on treatment with a specific CBD-based oil (see CBD-Based Oil Treatment section). Patients were recruited after voluntary, informed signed consent was provided by the legal guardian of the patients. We reviewed data obtained from medical charts and parents/caregivers' information. Clinical information included neurological examination, epilepsy type, age at seizure onset, seizure type, electroencephalographic (EEG) features, and drug treatments at baseline.

### Inclusion and exclusion criteria

Inclusion criteria were (1) age >1 year; (2) diagnosis of TRE as failing to achieve sustained seizure freedom with at least two antiepileptic drugs, tolerated and appropriately chosen; and (3) stable antiepileptic drug dosage for ≥4 weeks before CBD add-on. We excluded patients with (1) history of addiction or substance abuse; (2) previous use of any medical cannabis or CBD based products; and (3) incomplete documentation.

### CBD-based oil treatment

Patients were treated with an oral formulation of 24% CBD-based oil from *Cannabis Sativa*, produced by Enecta^®^. The hemp is grown from certified seeds following the EU regulation (council directive 2002/53EC), and each bottle contains 10 mL of hemp extract with 2400 mg of CBD (1 drop=7 mg of CBD).

Treatment was started at 5–10 mg/[kg·day] up to the maximum dose of 50 mg/[kg·day]; adjustments were made based on clinical response and tolerability. CBD-based oil was administered sublingually into three daily doses. CBD-based oil was always given at least 60 min after concomitant antiepileptic drugs to avoid drug–drug interactions. The baseline and minimum follow-up periods were 24 weeks. Seizure outcome was defined according to the following categories: seizure free, >50% improvement, <50% improvement, and no improvement/worsening. After the CBD-based oil treatment had reached the therapeutic dose, patients repeated a complete blood test (blood count, electrolytes, liver enzymes, bilirubin, and drug dosage).

### Statistical analysis

Statistical analysis was performed with MedCalc 9.2 (MedCalc Software, Mariakerke, Belgium) with a chi-square (*χ*^2^) statistic test's level of significance set at *p*<0.05.

## Results

### Clinical data

Thirty-seven patients met the inclusion criteria. Twenty (54%) patients were males, and the median age of participants was 16.1±12.3 years (range: 2–54 years). Demographic and clinical data of patients are summarized in Table 1. The average age at seizure onset was 3.8±2.1 years (range: 7 days–21 years).

**Table 1.** Demographic and Clinical Characteristics of the Patients

Clinical data	No. of participants
Male, *n* (%)	20 (54)
Female, *n* (%)	17 (46)
M:F	1:1.7
Average age at CBD treatment onset (years)	16.1±12.3
Average age at seizure onset (years)	3.8±2.1
Median therapeutic dosage (mg/[kg·day])	4.2±11.4
No. of AEDS at enrollment, *n* (%)	
≥4 AEDs	14 (38)
≥3 AEDs	9 (24)
≥2 AEDs	14 (38)
Seizure types, *n* (%)	
Focal motor seizures	17 (46)
Focal nonmotor seizures	4 (11)
Drop-attacks	5 (13)
Tonic–clonic seizures	11 (30)
Diagnostic categories, *n* (%)	
(Developmental) Epileptic encephalopathy	22 (60)
Focal epilepsy	9 (24)
Generalized epilepsy	6 (16)
Etiology, *n* (%)	
Acquired/lesional	11 (30)
Genetics	12 (32)
Unknown	14 (38)

AED, antiepileptic drug.

### Etiology of epilepsy and seizure types

A genetic etiology was demonstrated for 12 (32.4%) patients (*SCN8A*, *n*=3; *CDKL*5, *n*=2; *PCDH19*, *n*=1; *KCTN1*, *n*=1, *SCN1A*, *n*=1, *HEXA*, *n*=1; *TSC2*, *n*=1; *LIS1*, *n*=1; *dup14q11.2-q12; del2p;del15q11–q14*, *n*=1), acquired/lesional in 11 (30%) patients (hypoxic–ischemic brain damage, *n*=7; postinfectious brain damage, *n*=2; mesial temporal lobe epilepsy, *n*=1; FCD1, *n*=1) and unknown etiology in 14 (38%) patients. Twenty-two (60%) patients were diagnosed with (developmental) epileptic encephalopathy (i.e., in which seizures or epileptiform activity contributes to or exacerbates underlying brain dysfunction), nine (24%) patients had focal epilepsy, and six (16%) patients had generalized epilepsy. Seventeen (46%) patients suffer from focal motor seizures, 11 (30%) patients suffer from tonic–clonic seizures, 5 (13%) patients had drop attacks, and 4 (11%) patients suffer from focal nonmotor seizures.

### Efficacy data

**FIG. 1.**
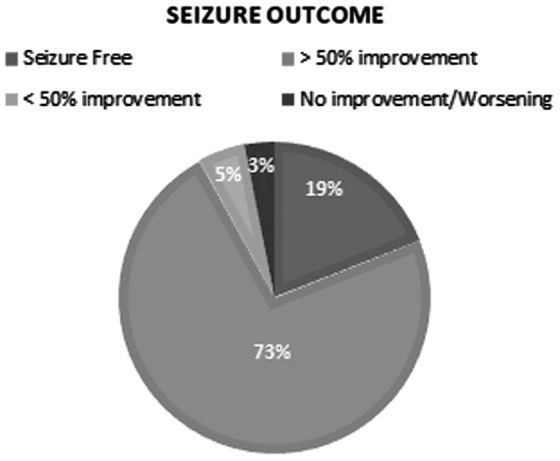
Seizure outcome in our cohort.

**FIG. 2.**
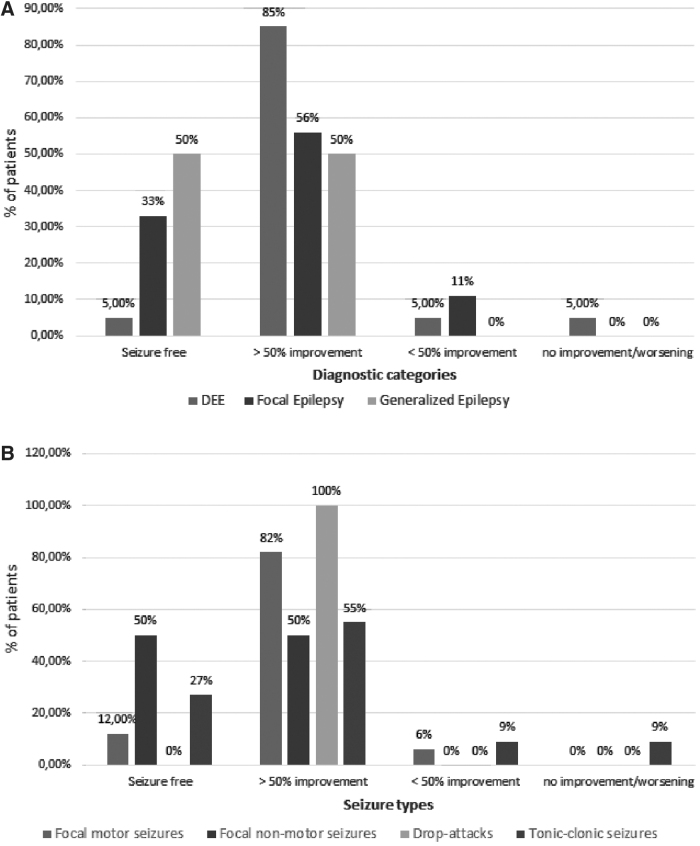
Seizure outcome according to diagnostic categories **(A)** and seizure types **(B)** in our cohort.

The median starting dose of CBD was 7.5 mg/[kg·day] (range: 5–10 mg/[kg·day]). The total median therapeutic dose achieved was 4.2±11.4 mg/[kg·day] (range: 0.6–50 mg/[kg·day]). Average follow-up duration was 68 weeks (range: 24–72 weeks). At the end of the trial, 7 (19%) patients became seizure free, 27 (73%) patients had a >50% improvement, and 2 (5%) patients showed a <50% improvement (Fig. 1). One (3%) patient discontinued CBD-based oil therapy due to lack of efficacy after 40 weeks. Seizure outcome according to diagnostic categories (A) and seizure types (B) in our cohort is shown in Fig. 2A, B. Among the 22 patients with epileptic developmental encephalopathy, 19 (85%) subjects showed an >50% improvement, 1 (5%) patient went seizure free, 1 (5%) subject had an improvement less than 50%, and another 1 (5%) had withdrawn due to the lack of efficacy. Among the nine patients with focal epilepsy, five (56%) had an improvement of 50% or more, one (11%) patient had an improvement less than 50%, and three (33%) patients became seizure free. Among the six patients with generalized epilepsy, three (50%) patients became seizure free and three (50%) had an improvement of at least 50%.

We found a correlation (*p*=0.026) between CBD efficacy (≥50% improvement) and epileptic encephalopathy (Fig. 2A). The five patients with drop seizures showed at least a 50% improvement. Fourteen (82%) out of the 17 patients with focal motor seizures had an improvement of 50% or more, 2 (12%) patients became seizure free, and 1 (6%) had an improvement less than 50% of this seizure type. Two (50%) out of 4 patients with focal nonmotor seizures had an improvement of 50% or more and 2 (50%) became seizure free; 6 (55%) out of 11 patients with tonic–clonic seizures had an improvement of 50% or more, 3 (27%) patients became seizure free, 1 (9%) had an improvement less than 50% of this seizure type, and 1 (9%) had withdrawn according to lack of efficacy (Fig. 2B). These data showed that in our cohort 27 (73%) out of 37 patients showed improvement in at least 50% of the seizure frequency. Notably, none of the patients reported worsening in seizure frequency.

We did not observe any correlation between CBD-based oil dose and seizure-free status (*p*=0.9) or between the maximum dose of CBD-based oil and both groups with ≥or <50% improvement (*p*=0.86). Moreover, no significant difference was found for CBD-based oil efficacy in different seizure types (*p*=0.13). In our cohort, the average number of concomitant antiepileptic drugs taken at baseline period was 4 (range: 1–11); 14 (38%) patients took ≥4 antiepileptic drugs; 9 (24%) patients took >3 (38%) antiepileptic drugs; and 14 patients took >2 antiepileptic drugs. Twenty-four out of 37 took valproic acid (*n*=18; 49%) and clobazam (*n*=6; 16%). There was no difference in the efficacy of CBD in the four (16%) individuals receiving the acid valproic/clobazam combination therapy (*p*=0.54) versus with the other patients from our cohort. Moreover, none of the seizure-free patients did receive a combination of valproic acid and clobazam.

**FIG. 3.**
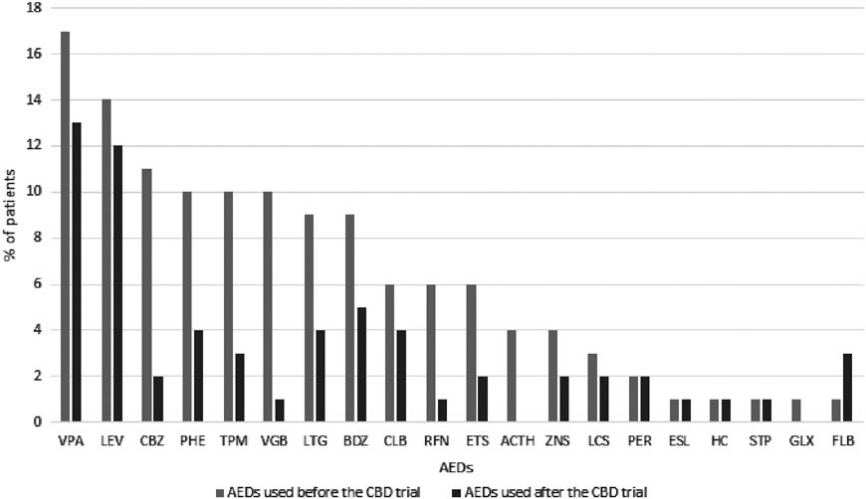
Antiepileptic drugs used before (blue bar) and after (orange bar) the CBD trial. VPA, Valproic Acid; LEV, Levetiracetam; CBZ, Carbamazepine; PHE, Phenytoin; TPM, Topiramate; VGB, Vigabatrin; LTG, Lamotrigine; BDZ, Benzodiazepines; CLB, Clobazam; RFN, Rufinamide; ETS, ethosuximide; ZNS, zonisamide; LCS, Lacosamide; STP, Stiripentol; FLB, Felbamate.

Patients with at least a 50% reduction of seizures reduced the dose of concomitant drugs 24 weeks after the CBD-based oil introduction. At 40 weeks follow-up, 10 patients (27%) were on seizure control in association with only one drug (Fig. 3).

### Safety and tolerability

None of our patients did report seizure worsening/status epilepticus. Mild and temporary adverse effects were reported in 9 (25%) out of 37 patients, including somnolence in 7 patients (77%) and loss of appetite in 2 patients (23%). Four (57%) out of seven patients with somnolence were on valproic acid/clobazam combination therapy but we did not observe any change in blood and laboratory examinations, including liver function enzymes, as well as therapeutic drug monitoring levels, at the end of the follow-up period, and no dose modification of CBD or concomitant drugs was applied (data not shown).

## Discussion

This study showed the efficacy of a 24% CBD-based oil formulation with few significant side effects in patients with TRE of various etiologies. We observed at least 50% seizure improvement in 73% of patients and a seizure freedom in 19% of the population. Only one patient withdrew because of lack of efficacy, and no patient did report seizure worsening after the introduction of CBD-based oil.

This “real-world” study further supports the efficacy of CBD-based preparations in various epilepsy types and etiologies.^21,23,27^ We did not mainly focus on the efficacy of CBD as there are already strong data on Epidiolex^®^ in DS, LGS, and Tuberous Sclerosis Complex coming from placebo-controlled trials with robust numbers and rigorous follow-up. However, many individuals do not have access to the FDA approved compound and are using various CBD preparations available without a prescription. During the follow-up period, which extended from 24 to 72 weeks, 36/37 patients remained in the study. This is possibly due to the maintenance of long-term efficacy and mild adverse events in our cohort.

Several authors reported similar beneficial effects in various open-label trials with other formulations/preparations.^10,21,28,29^ The reported median dosage applied in our cohort (4.2 mg/kg) is far below any of those found effective in other studies with CBD. The bioavailability of oral CBD is around 10%, whereas the sublingual bioavailability is at least ≥80%.^17–19^ Therefore, the sublingual administration of CBD in our patients could be the main explanation for this unusual finding although we cannot exclude that it is related to the intrinsic features of epilepsy in our series or to other factors.

Additional markers of efficacy included the observed weaning from other antiepileptic drugs. In our study, patients were on two or more antiepileptic drugs (median: 4 drugs) at baseline. Following the introduction of CBD-based oil, after at least 24 weeks, the reduction of other concomitant antiepileptic drugs was observed, and these data are consistent with those reported in other previous studies.^21,22,30^ In the following weeks, 10 (27%) of all our patients were able to interrupt almost all the other antiepileptic drugs, and although they did not end up in CBD-based oil monotherapy still seizures were controlled with only one antiseizure medication over CBD. The most used concomitant drug was valproic acid.

A recent study demonstrated a clinical interaction of CBD with clobazam. Indeed, CBD inhibits the catabolism of its active metabolite, *N*-desmethylclobazam, and this may explain why patients taking both antiepileptic drugs are more likely to experience sedation.^31^ These side effects were alleviated with reduction of clobazam dose with no change in the efficacy.^32^ Other studies reported elevated transaminase levels in 1–23% of children receiving CBD in the add-on to valproic acid.^33,34^ The other most common side effects reported include gastrointestinal-related adverse events (diarrhea, vomiting, and decreased appetite/weight loss). In our study, 9 of the 24 (37.5%) patients on clobazam and/or valproic acid showed adverse events, that is, somnolence or loss of appetite.

CBD exposure is increased by five times when this drug is taken with high-fatty foods.^6,35^ However, due to the sublingual administration of CBD in this trial, we did not methodically collect data on food intake in our patients. Due to the open retrospective nature of the study, drugs' levels before and after the CBD treatment were also not methodically collected. However, no dose reduction of CBD or concomitant drugs was necessary/observed, neither alterations in laboratory parameters were observed in our cohort.

Our results are in line with another open-label study in TRE of different etiologies.^24^ Devinsky et al.^19,24^ evaluated the efficacy of an oral pharmaceutical formulation of highly purified CBD (Epidyolex) in DS and LGS and showed a reduction of 36.5% in tonic seizures and 16% in tonic–clonic seizures. Thiele et al.^36^ found that LGS patients receiving add-on oral formulation of Epidyolex experienced a ≥50% reduction in monthly drop seizure frequency compared with 24% in those receiving placebo. Moreover, they did observe that treatment with CBD reduced the median frequency of total seizures and nondrop seizures compared to placebo.^37^ Similarly, in our cohort, add-on of CBD induced a significant improvement (>50%) of all seizure types, although the efficacy was better on both focal motor seizures (88%) and drop seizures (100%). Otherwise, data on the literature showed^19,24,35,36,38,39^ the lack of a significant effect on nonconvulsive seizure frequency, suggesting that the antiseizure effect of CBD may be specific to convulsive seizures but these data are more difficult to evaluate in developmentally delayed children. Moreover, in our study, the clinical response seemed to be not correlated with higher CBD dose. Indeed, there was no statistically significant difference in maximum daily CBD dose between the group of patients who showed ≥50% improvement and the group that showed <50% improvement (*p*=0.86). In our series, CBD-based oil was well tolerated in most patients, regardless of etiology. However, these results may be applied to these specific products and may not be directly transferred to other products considering that the extract also contains other substances, which may possess their efficacy.

### Limitations of the study

Limitations of this study include the potential bias for patients' selection, the drug schedule administration, as well as the assessment of the CBD efficacy based on the clinical outcome rather than on EEG findings. In addition, in this trial, therapeutic drug monitoring of blood CBD levels was not available, adjustments of the add-on CBD dose were made based on clinical response and tolerability (flexible titration schedule), and the efficacy of the drug was assessed by responder categories and seizure-free rates. Moreover, we included a heterogeneous group of patients of different ages and etiologies of epilepsy, and most patients with developmental epileptic encephalopathy featured multiple seizures; in these patients, efficacy was assessed on the prevalent and most impactful seizure type reported by caregivers and clinicians. Finally, due to the retrospective nature of the study, we could not assess the time course of the efficacy of CBD add-on treatment. Moreover, adult patients used a higher number of antiepileptic drugs before the introduction of CBD-based oil, related to a significantly longer duration of epilepsy than children.

## Abbreviations Used

AED: antiepileptic drugs
CBD: cannabidiol
DS: Dravet Syndrome
EEG: electroencephalogram
LGS: Lennox–Gastaut syndrome
THC: tetrahydrocannabinol
TRE: treatment-resistant epilepsy
